# Biochemical Properties of Polyphenol Oxidases from Ready-to-Eat Lentil (*Lens culinaris* Medik.) Sprouts and Factors Affecting Their Activities: A Search for Potent Tools Limiting Enzymatic Browning

**DOI:** 10.3390/foods8050154

**Published:** 2019-05-07

**Authors:** Małgorzata Sikora, Michał Świeca, Monika Franczyk, Anna Jakubczyk, Justyna Bochnak, Urszula Złotek

**Affiliations:** Department of Biochemistry and Food Chemistry, University of Life Sciences, Skromna Str. 8, 20-704 Lublin, Poland; malgorzata.sikora@up.lublin.pl (M.S.); monika.franczyk@up.lublin.pl (M.F.); anna.jakubczyk@up.lublin.pl (A.J.); just.bochnak@gmail.com (J.B.); urszula.zlotek@up.lublin.pl (U.Z.)

**Keywords:** biochemical characteristic, enzymatic browning, inhibitory profile, lentil, sprouts, polyphenol oxidase, purification

## Abstract

Enzymatic browning of sprouts during storage is a serious problem negatively influencing their consumer quality. Identifying and understanding the mechanism of inhibition of polyphenol oxidases (PPOs) in lentil sprouts may offer inexpensive alternatives to prevent browning. This study focused on the biochemical characteristics of PPOs from stored lentil sprouts, providing data that may be directly implemented in improving the consumer quality of sprouts. The purification resulted in approximately 25-fold enrichment of two PPO isoenzymes (PPO I and PPO II). The optimum pH for total PPOs, as well as for PPO I and PPO II isoenzymes, was 4.5–5.5, 4.5–5.0, and 5.5, respectively. The optimal temperature for PPOs was 35 °C. Total PPOs and the PPO I and PPO II isoenzymes had the greatest affinity for catechol (*K_m_* = 1.32, 1.76, and 0.94 mM, respectively). Ascorbic acid was the most effective in the inhibition of dark color formation by total PPOs, and showed ca. 62%, 43%, and 24% inhibition at 20-, 2-, and 0.2-mM concentrations. Ascorbic acid, l-cysteine, and sodium metabisulfite (20 mM) significantly inhibited color development in the reactions catalyzed by both isoenzymes of PPO. Ba^2+^, Fe^3+^, and Mn^2+^ (10 mM) completely inhibited PPO activity. This study of the effect of antibrowning compounds and cations on PPO activity provides data that can be used to protect lentil sprouts against enzymatic browning during storage and processing.

## 1. Introduction

Polyphenol oxidases (PPOs) (EC 1.14.18.1, EC 1.10.3.1, or EC 1.10.3.2) are widely distributed in the plant kingdom, and their level and activity are dependent on the age, species, variety, maturity, and stress status of plants [1,2]. In addition, they are located in certain organelles, such as chloroplast thylakoids, peroxisomes, and mitochondria [1]. According to substrate specificity, three main types of phenol oxidases are known: (I) Monophenol monooxygenase (also called tyrosinase, monophenol oxidase, or cresolase) catalyzes the hydroxylation of monophenol to ortho-diphenol and the oxidation of diphenol to ortho-quinone; (II) diphenol oxidase (also called catechol oxidase, polyphenol oxidase, or *o*-diphenolase) catalyzes the oxidation of ortho-phenol, but cannot catalyze the oxidation or monooxygenation of metaphenol and para-phenol; and (III) laccase catalyzes the oxidation of ortho-phenol and para-phenol, but cannot catalyze the oxidation of monophenol and metaphenol [3]. This classification, although commonly used, also has some inaccuracies, e.g., in the case of mung bean [4] or tobacco [5] laccases, which share many substrates with PPOs. Enzymatic browning of plant-derived foods (including sprouts) contributes to a decrease in the sensory properties and marketability of fruits and vegetables [6,7,8,9]. The formation of brown or black pigments is due to increased activity of PPOs, resulting in the polymerization of quinones [2,7]. Additionally, increased activity of PPOs can decrease the level of phenolic compounds, i.e., plant secondary metabolites with well-documented nutraceutical properties [10,11]. Due to these facts, the characterization of PPO activities or the removal of reactants such as oxygen and phenolic compounds, especially those concerning potential inhibitors, are of increasing interest in the food industry. As one of the antibrowning agents, ascorbic acid inactivates PPOs irreversibly in the absence of PPO substrates, probably through binding to its active site, preferentially in its oxy form. Additionally, it can reduce reaction products, limiting the formation of a dark color. Cysteine activity is usually attributed to various mechanisms, e.g., its nucleophilic reactivity toward quinones to give a colorless adduct or its ability to reduce *o*-quinones to their polyphenol precursors. Citric acid and ethylenediaminetetraacetic acid sodium salt (EDTA) chelate copper at an enzyme-active site [12,13].

Polyphenol oxidase has been widely studied in fruits, vegetables, and mushrooms, e.g., eggplant (*Solanum melongena*) [14], persimmon [15], broccoli (*Brassica oleracea* var. *botrytis italica*) [16], celery [17], and butter lettuce (*Lactuca sativa* var. *capitata* L.) [18]: However, there are very few data that have presented the characterization of PPOs from edible sprouts. 

In this paper, we report the isolation, partial purification, and biochemical properties of two isoenzymes and total PPO activity in lentil sprouts (*Lens culinaris* Medik.). Special attention is placed on the factors affecting PPO activity, which may be useful for protecting sprouts against PPO-related undesirable changes in their quality.

## 2. Materials and Methods

### 2.1. Chemicals

Catechol, Diethylaminoethyl–Sepharose (DEAE–S), tris(hydroxymethyl)aminomethane (TRIS), ethylenediaminetetraacetic acid sodium salt (EDTA), 4-methylcatechol, gallic acid, caffeic acid, l-cysteine, ascorbic acid, and dl-dithiothreitol were obtained from Sigma-Aldrich (Poznań, Poland). All other chemicals were of analytical grade.

### 2.2. Materials and Sprouting Conditions

Seeds from the lentil cultivar Tina were purchased from PNOS S.A. Ozarów Mazowiecki (Poland). The seeds were sterilized in 10% (*v/v*) sodium hypochloride for 10 min, drained, and washed with distilled water until they reached a neutral pH. They were placed in distilled water and soaked for 6 h at 25 °C. The seeds were dark-germinated for 4 days in a growth chamber on Petri dishes (diameter, 125 mm) lined with absorbent paper. Seedlings were watered daily with 5 mL of Milli-Q water [19]. 

### 2.3. Enzyme Assay

Polyphenol oxidase (PPO) activity was determined by measuring the initial rate of quinone formation, as indicated by an increase in the absorbance units (AUs) at 420 nm. An increase in absorbance of 0.001 min^−1^ was taken as one unit of enzyme activity [20]. The increase in absorbance was linear with time for the first 120 s. The sample contained 1 mL of a 0.05-M substrate solution prepared in TRIS-HCl buffer (50 mM, pH 6.5) and 0.05 mL of an enzyme solution. All measurements were performed in triplicate.

### 2.4. Protein Determination

Protein content was determined according to the dye-binding method proposed by Bradford [21] using bovine serum albumin as a standard.

### 2.5. Enzyme Extraction and Partial Purification

One-hundred grams of sprouts were homogenized in 250 mL of 50 mM TRIS-HCl buffer (pH 6.5) containing 10 mM of ascorbic acid and 0.5% polyvinylpyrrolidone and were extracted with the aid of a magnetic stirrer for 1 h at 4 °C. The crude extract samples were centrifuged at 9000× *g* for 20 min at 4 °C. Solid (NH_4_)_2_SO_4_ was added to the supernatant to obtain 80% saturation. After that, the precipitated proteins were separated by centrifugation at 9000× *g* for 30 min at 4 °C. The precipitate was dissolved in 60 mL of 5-mM TRIS-HCl (pH 7.0) and was dialyzed for 48 h using the same buffer in a cellulose bag with a membrane MWCO bigger than 12,000 Da at 4 °C. Afterwards, the dialysate was transferred to a DEAE–Sepharose column (20 × 250 mm) equilibrated with 5 mM of TRIS-HCl buffer, pH 6.5. Proteins were eluted, employing a linear gradient of 0 to 1.0 M of NaCl in 5 mM of TRIS-HCl buffer (pH 6.5) at a 30-mL·h^−1^ flow rate. Three-milliliter fractions were collected, for which protein content (280 nm) and PPO activity toward catechol as a substrate were monitored. Fractions that showed PPO activity were collected. 

### 2.6. Characterization of PPO

#### 2.6.1. Kinetic Data Analysis and Substrate Specificity

The specificity of PPOs from the lentil sprout extract was investigated for five commercial grade substrates (catechol, 4-methylcatechol, gallic acid, caffeic acid, and (+)-catechin) at concentrations of 1, 5, 10, 20, and 30 mM. The Michaelis constant (*Km*), maximum reaction velocities (*V_max_*), and specificity (*V_max_*/*Km*) of the PPOs were determined with the Lineweaver–Burk method.

#### 2.6.2. Effect of Temperature on Enzyme Activity

PPO activity was determined as a function of temperature in standard conditions at a temperature range of 20–80 °C. The optimum temperature for the PPO was determined using 50 mM of catechol as a substrate. The substrate solution was heated to the tested temperature, and then the enzyme solution was added. PPO activity was calculated in the form of percent residual PPO activity at the optimum temperature.

#### 2.6.3. Effect of pH on Enzyme Activity

PPO activity was determined as a function of pH in standard conditions using various buffers in the pH buffering range of 3.5–8.0 (3.5–5.5 acetate buffer, 100 mM; 5.5–8.0 potassium phosphate buffer, 100 mM). The optimum pH for the PPO was determined using 0.05 M of catechol as a substrate. The pH value corresponding to the highest enzyme activity was taken as the optimal pH. PPO activity was calculated in the form of residual PPO activity at the optimum pH. 

#### 2.6.4. Effect of Antibrowning Agents on PPOs

The effects of ascorbic acid, citric acid, EDTA, l-cysteine, sodium azide, dithiothreitol, and sodium metabisulfite on PPO activity were examined. Three different concentrations of these inhibitors (0.2, 2, and 20 mM) were tested using 50 mM of the catechol substrate and were compared to a control enzyme reaction performed in optimal conditions with no inhibitor added. Percentage inhibition was calculated using the following equation:

Inhibition (%) = (*A*_0_ − *A_i_*/*A*_0_) × 100%, (1)

where *A*_0_ is initial PPO activity (without the inhibitor), and *A_i_* is PPO activity with the inhibitor.

#### 2.6.5. Effect of Ions on Enzyme Activity

The effect of ions, including Na^+^, K^+^, Mg^2+^, Zn^2+^, Ba^2+^, Fe^3+^, and Mn^2+^ (chloride salts), on PPO activity was determined. Two different concentrations of these cations (2 and 10 mM) were tested using 50 mM of the catechol substrate. The effect of the studied ions on PPO activity was calculated in the form of percent residual PPO activity in comparison to the nontreated enzyme preparation.

### 2.7. Statistical Analysis

All data are presented as means including standard deviations (SDs) of three assays (means ± SD, *n* = 3).

## 3. Results and Discussion

### 3.1. PPO Isolation and Partial Purification

**Figure 1:**
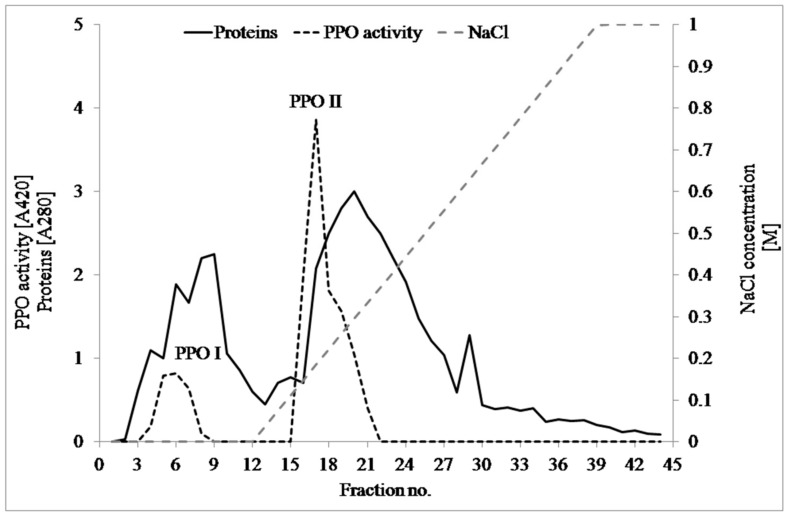
Anion exchange chromatographic elution profiles obtained after applying dissolved and desalted saline precipitate extract of lentil sprouts.

PPO was partially purified using a combination of ammonium sulfate precipitation and ion exchange chromatography (Figure 1). Two isoenzymes of PPO were found: PPO I and PPO II. The results of the purification of PPO are given in Table 1. After ammonium sulfate precipitation, the yield and purification fold were 90.6% and 4.67, respectively. The purification folds after ion exchange chromatography were 26.1 and 25.11 for the first and second isoenzymes, respectively. Further biochemical studies were performed on the first and second isoenzymes (important data in the enzymology field) and the total (crude extract) PPOs (data for food technology). 

Catechol was used as a substrate for measuring PPO activity. An increase in absorbance of 0.001 min^−1^ was taken as one unit of enzyme activity.

### 3.2. Substrate Specificity and Some Kinetic Parameters of Lentil Sprout PPOs

PPO kinetics were studied with four substrates, those commonly used for PPO assays (catechol, 4-methylcatechol) as well as those that are important from the nutraceutical point of view (gallic and caffeic acids). The *K_m_* and *V_max_* values calculated from the Lineweaver–Burk graphs are shown in Table 2. The values of *V_max_* and catalytic efficiency (*V_max_*/*K_m_*) indicated that 4-methylcatechol was the most suitable phenolic substrate for lentil sprout PPOs (Table 2). The *V_max_* values of total PPOs as well as PPO I and PPO II isoenzymes against gallic acid were also very high, but the *K_m_* of total PPOs was nearly twice and three times higher than the first and second isoenzymes. Most importantly, only PPO I used caffeic acid as a substrate (*K_m_* = 3.8 mM, *V_max_* = 769 U·mL^−1^·min^−1^). Total PPOs as well as PPO I and PPO II had the greatest affinity for catechol (*K_m_* = 1.32, 1.76, and 0.94 mM, respectively). These values were lower than those previously determined for persimmon (*K_m_* = 25 mM; sodium acetate buffer, pH 5.5) [15], green beans (*K_m_* = 10.6 and 37 mM for PPOI and PPOII, respectively; phosphate buffer, pH 7.0) [22], and mango fruit (*K_m_* = 10.6 mM, sodium acetate buffer, pH 5.6) [23]. All the studied PPOs of lentil sprouts exhibited the highest affinity for catechol: Hence, it was used as a substrate in further biochemical assays. 

### 3.3. Effect of Temperature and pH on PPO Activity

**Figure 2:**
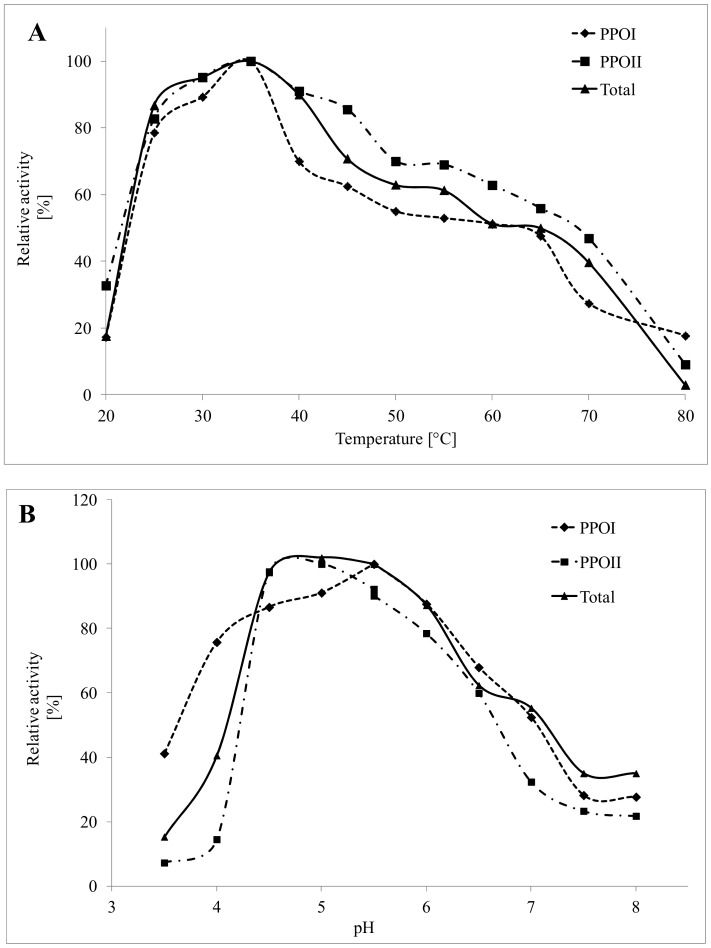
Effects of temperature (**A**) and pH (**B**) on the activity of lentil sprout PPOs.

Figure 2A shows the influence of temperature on PPO activities in the assay conditions (pH 5.5 and 50 mM catechol as a substrate). PPO I, PPO II, and total PPOs reached maximum activity at 35 °C. The optimal temperatures for PPO activity are substrate-dependent and may differ for PPOs obtained from various sources [24]. It has been reported that when catechol is used as a substrate, the optimum temperature is 40 °C for PPOs from Chinese cabbage [25], soybean sprouts [26], and parsley [24]; and 25–30 °C for bananas [27]. Higher optimal temperatures were reported by Serradell et al. [28] and Navarro et al. [15] for PPOs isolated from strawberries (50 °C) and persimmons (55 °C).

The assay of PPO activity in a pH range from 4.0 to 8.0, using catechol as a substrate, showed optimal conditions for total PPO activity in the pH range of 4.5–5.5. When the two PPO isoforms were studied separately, a pH of 4.5–5.0 and 5.5 were found to be optimal for PPO I and PPO II, respectively (Figure 2B). The pH optimum for PPO activity has been found to be dependent on the enzyme source and purity, substrate, and buffer system used [1]. In the available literature, different pH optima for PPO activity have been reported. Two pH optima, suggesting the presence of two PPO isoenzymes, have been previously reported by other researchers, i.e., 4.5–5.0 and 7.5–7.6, for two PPO isoenzymes from avocados [29]; 4.0 and 7.0 for green bean PPOs [22]; and 5.0 and 7.5 for Jonagored apple PPOs [30]. A pH value of 5.5, i.e., the optimum pH for PPO activity determined in this study, agrees well with values that have been reported for lettuce [18] and mango fruit [23]. Contrarily, in a study performed by Nagai and Suzuki [26], PPOs from soybean sprouts exhibited optimal activity at pH 8–9.

### 3.4. Effect of Various Inhibitors and Metal Ions on PPO Activity

The effects of antibrowning agents on the activity of PPOs from lentil sprouts were studied using catechol as a substrate. The results, i.e., the percentage of inhibition relative to the control, are reported in Table 3. Ascorbic acid was the most effective inhibitor of total PPOs and showed ca. 62%, 43%, and 24% of inhibition of dark pigment formation at 20-mM, 2-mM, and 0.2-mM concentrations, respectively. The production of dark quinones by both isoenzymes was most effectively inhibited by 20 mM of ascorbic acid, l-cysteine, and sodium metabisulfite. These compounds also showed high efficiency at the lowest concentrations studied. Similar profiles have been previously found for PPOs from bananas [31], parsley [24], green beans [22], mango fruit [23], and soybean sprouts [26]. PPO II was also very sensitive to citric acid: Approximately 60% inhibition was recorded in the presence of 20 mM of the inhibitor. Furthermore, the purified PPOs were much more sensitive to the studied inhibitors than the crude extract, probably because some components were able to mask the inhibitory effect of these compounds. The degree of dark pigment formation depends on the origin of the PPO and substrate used, and thus it is difficult to compare the present results to other studies. Yagar and Sagiroglu [32] recorded 98% and 100% of inhibition of quince PPOs in the presence of 2 and 20 mM of ascorbic acid, respectively. For 2 and 20 mM of sodium metabisulfite, the degree of inhibition was 52% and 98%, respectively. As in other reports of the effect of antibrowning agents [11,20,33,34], the activity of lentil sprout PPOs was also inhibited by a thiol-containing compound (dithiothreitol) and a copper-binding ligand (sodium azide, EDTA), but these compounds are very toxic and are banned as food additives. Thus, of the studied antibrowning agents, only ascorbic acid, citric acid, and l-cysteine are suitable to be used in food technology.

The effect of metal ions on the activity of PPOs is presented in Table 4. Zn^2+^, Ba^2+^, Fe^3+^, and Mn^2+^ at a 10-mM concentration completely inhibited the activity of PPOs. In contrast, in studies conducted by Liu et al. [35], both Zn^2+^ and Mg^2+^ (10 mM) increased the activity of PPOs from flower buds of *Lonicera japonica* by about 10%–15%. In addition, Aydemir has reported [36] that the activity of PPOs from artichokes was only slightly inhibited by Zn^2+^ and Mg^2+^ at 1- and 10-mM concentrations (Fe^3+^ did not affect activity). Total PPOs, PPO I, and PPO II were inhibited by 2 mM of MgCl_2_: 50%, 52%, and 67% of residual activity was detected, respectively. According to literature data, the effect of Mg^2+^ on the activity of PPOs differs significantly and is strongly determined by the origin of the PPOs. The activity of PPOs from flower buds of *Lonicera japonica* has been activated by ions at 1–100 mM concentrations [35], whereas reduced activity has been noted at lower concentrations of ions (0.1–0.01 mM). An opposite relationship has been observed for PPOs from green beans [22] and artichokes [36]. There was no effect of 10 mM of K^+^ on the activity of PPOs (except PPO I): However, at the 2-mM concentration, these ions activated PPO I, PPO II, and total PPOs. A similar pattern of relationships was recorded for total PPOs, PPO I, and Na^+^ ions. It has been previously reported that Na^+^ ions either did not affect or only slightly modified the activity of PPOs, e.g., from green beans [22], flower buds of *Lonicera japonica* [35], or artichokes [36]. On the other hand, the activities of PPOs from Ataulfo mango and Anamur banana have been inhibited by Na^+^ ions [18,21].

## 4. Conclusions

Lentil sprouts are widely consumed all over the world. Enzymatic browning of sprouts during storage is a serious problem negatively influencing their consumer quality. Identifying and understanding the mechanism of inhibition of polyphenol oxidases (PPOs) in lentil sprouts may offer inexpensive alternatives in preventing browning. Our findings indicated that supplementation of sprouts with metal ions (Zn^2+^, Mn^2+^, Fe^3+^) and/or inhibitors (ascorbic acid, citric acid) may be used for decreasing the activity of PPOs. This strategy seems to be justified, but more research is needed to define effects on the growth and metabolism of sprouts, as well as their nutritional and pro-health qualities.

## Figures and Tables

**Table 1:** Purification chart of polyphenol oxidases (PPOs) from lentil sprouts.

		Total Volume (mL)	Activity (U/mL)	Protein (mg/mL)	Total Activity (U)	Specific Activity (U/mg)	Yield (%)	Purification Fold
Crude extract		250	550	732.00	137500	0.75	100.0	1.00
Salting out and dialysis		55	2265	644.97	124575	3.51	90.60	4.67
Ion exchange chromatography	PPO I	13	1815	92.56	23595	19.61	17.16	26.10
	PPO II	19	4475	237.12	85016	18.87	61.83	25.11

**Table 2:** Kinetic parameters of PPOs from lentil sprouts assessed with the use of several phenol substrates.

		*V_max_* (U·mL^−1^·min^−1^)	*Km* (mM)	*V_max_*/*Km* (U·mL^−1^·min^−1^·mM^−1^)
4-methylcatechol	PPO I	4878 ± 244	3.00 ± 0.14	1626
	PPO II	3846 ± 192	3.40 ± 0.15	1131
	Total	5410 ± 270	1.50 ± 0.07	3607
Catechol	PPO I	952 ± 48	1.76 ± 0.08	541
	PPO II	1111 ± 56	0.94 ± 0.04	1176
	Total	1737 ± 87	1.32 ± 0.06	1320
Gallic acid	PPO I	2817 ± 141	2.25 ± 0.10	1250
	PPO II	3742 ± 152	5.00 ± 0.23	769
	Total	8250 ± 413	7.25 ± 0.33	1138
Caffeic acid	PPO I	769±38	3.81 ± 0.17	202
	PPO II	0	0	0
	Total	0	0	0

All values represent the means of triplicate measurements.

**Table 3:** Effects of various antibrowning agents on the activity of lentil sprout PPOs.

	Concentration of Compounds (mM)	% of Inhibition		
		PPO I	PPO II	Total
Ascorbic acid	20	79.66 ± 3.03	79.42 ± 1.80	62.57 ± 2.38
	2	50.85 ± 1.93	59.42 ± 2.58	43.86 ± 1.67
	0.2	46.61 ± 1.77	62.32 ± 0.71	24.56 ± 0.93
l-cysteine	20	71.67 ± 2.72	72.09 ± 3.14	26.97 ± 1.02
	2	68.33 ± 2.60	55.81 ± 2.43	21.35 ± 0.81
	0.2	56.67 ± 2.15	34.88 ± 1.52	10.11 ± 0.38
Na_2_S_2_O_5_	20	76.03 ± 2.89	71.58 ± 3.11	25.88 ± 0.98
	2	56.20 ± 2.14	64.21 ± 2.79	14.12 ± 0.54
	0.2	18.18 ± 0.69	24.21 ± 1.05	1.18 ± 0.04
EDTA	20	26.67 ± 1.01	39.77 ± 1.73	14.63 ± 0.56
	2	24.17 ± 0.92	36.36 ± 1.58	3.66 ± 0.14
	0.2	20.83 ± 0.79	34.09 ± 1.48	2.44 ± 0.09
Citric acid	20	30.13 ± 1.14	60.82 ± 2.65	43.18 ± 1.64
	2	22.44 ± 0.85	54.39 ± 2.37	37.88 ± 1.44
	0.2	7.69 ± 0.29	57.89 ± 2.52	8.33 ± 0.32
Sodium azide	20	22.50 ± 0.85	22.89 ± 1.00	9.09 ± 0.35
	2	17.50 ± 0.67	15.66 ± 0.68	8.08 ± 0.31
	0.2	5.83 ± 0.22	8.43 ± 0.37	2.02 ± 0.08
Dithiothreitol	20	17.50 ± 0.67	37.89 ± 1.65	30.26 ± 1.15
	2	22.50 ± 0.86	31.58 ± 1.37	13.16 ± 0.50
	0.2	22.50 ± 0.86	15.58 ± 1.72	1.32 ± 0.05

All values represent the means of triplicate measurements.

**Table 4:** Effects of metal ions on the activity of PPOs from lentil sprouts.

Ion Concentration (mM)		Residual Activity (%)	
		10	2
Na^+^	PPO I	75.82 ± 3.26	82.42 ± 3.54
	PPO II	90.38 ± 3.89	142.31 ± 6.12
	Total	89.81 ± 3.86	106.48 ± 4.58
K^+^	PPO I	76.99 ± 3.31	118.58 ± 5.10
	PPO II	101.35 ± 4.36	159.46 ± 6.86
	Total	99.23 ± 4.27	109.46 ± 4.71
Mg^2+^	PPO I	46.90 ± 2.02	50.44 ± 2.17
	PPO II	51.35 ± 2.21	52.70 ± 2.27
	Total	61.54 ± 2.65	67.69 ± 2.91
Zn^2+^	PPO I	Nd	42.24 ± 1.82
	PPO II	Nd	38.42 ± 1.65
	Total	Nd	48.78 ± 2.10
Ba^2+^	PPO I	Nd	54.23 ± 2.33
	PPO II	Nd	54.43 ± 2.34
	Total	Nd	86.05 ± 3.70
Fe^3+^	PPO I	Nd	53.49 ± 2.30
	PPO II	Nd	56.96 ± 2.45
	Total	Nd	58.28 ± 2.51
Mn^2+^	PPO I	Nd	48.98 ± 2.11
	PPO II	Nd	32.92 ± 1.42
	Total	Nd	27.30 ± 1.17

All values represent the means of triplicate measurements. Nd: not detected.
